# Antibacterial and Antioxidant Activities of Essential Oils from *Artemisia herba-alba* Asso., *Pelargonium capitatum × radens* and *Laurus nobilis* L.

**DOI:** 10.3390/foods5020028

**Published:** 2016-04-11

**Authors:** Ragina Rafiq, Saeed A. Hayek, Ugochukwu Anyanwu, Bonita I. Hardy, Valerie L. Giddings, Salam A. Ibrahim, Reza Tahergorabi, Hye Won Kang

**Affiliations:** Food and Nutritional Sciences, Department of Family and Consumer Sciences, North Carolina Agricultural and Technical State University, Greensboro, NC 27411, USA; rrafiq@aggies.ncat.edu (R.R.); safesaeed@yahoo.com (S.A.H.); ucanyanw@aggies.ncat.edu (U.A.); bimilfor@ncat.edu (B.I.H.); vlgiddin@ncat.edu (V.L.G.); ibrah001@ncat.edu (S.A.I.); rtahergo@ncat.edu (R.T.)

**Keywords:** rose geranium, white wormwood, bay laurel, antioxidant properties, antibacterial activities

## Abstract

Essential oils are natural antimicrobials that have the potential to provide a safer alternative to synthetic antimicrobials currently used in the food industry. Therefore, the aim of this study was to evaluate the antimicrobial and antioxidant activities of essential oils from white wormwood, rose-scented geranium and bay laurel against *Salmonella typhimurium* and *Escherichia coli* O157:H7 on fresh produce and to examine consumer acceptability of fresh produce treated with these essential oils. Our results showed that essential oil derived from rose-scented geranium exhibited the most effective antimicrobial activity at the same and similar minimum inhibition concentration levels (0.4%, *v*/*v* and 0.4% and 0.5%, *v*/*v*) respectively against *Salmonella typhimurium* and *Escherichia coli* O157:H7. All three essential oils showed antioxidant properties, with the highest activity occurring in bay laurel essential oil. In a sensory test, tomatoes, cantaloupe and spinach sprayed with 0.4% rose-scented geranium essential oil received higher scores by panelists. In conclusion, rose-scented geranium essential oil could be developed into a natural antimicrobial to prevent contamination of *Salmonella typhimurium* and *Escherichia coli* O157:H7 in fresh produce, plus this oil would provide additional health benefits due to the antioxidant properties of its residue.

## 1. Introduction

Foodborne illnesses are an increasingly common public health problem around the world [1]. These illnesses are generally caused by the consumption of foods contaminated with pathogenic microorganisms during different stages of pre- and post-harvest processing. Although various pathogens have been associated with the occurrence of foodborne illness, *Escherichia coli (E. coli)* O157:H7, *Listeria* and *Salmonella* are the most common pathogens related to multi-state outbreaks in the United States. Among these pathogens, *Salmonella typhimurium (S. typhimurium)* and *E. coli* O157:H7 were more likely to contaminate meat and fresh produce in the U.S. [2,3].

A variety of synthetic antimicrobials have been used to reduce bacterial contamination in meat and fresh produce. However, synthetic antimicrobials have been associated with health problems such as hypersensitivity, allergies, asthma, hyperactivity and cancer [4]. These effects may be due to the direct side effects of food consumption containing antimicrobials during long- or short-term periods or due to the presence of residues of antimicrobials on foods [4]. In addition, synthetic antimicrobials can kill both bad and good bacteria in the human intestine [5], which could result in negative health consequences for consumers. Another concern related to the use of synthetic antimicrobials is an increased bacterial resistance to antimicrobials [6]. Because some bacterial strains develop protective systems and mechanisms to avoid antimicrobial effects, new drug-resistant bacterial strains can thrive [6]. Due to the growing health concerns by consumers, microbial susceptibility to resistance, and industrial interest in developing low cost and more effective agents, natural resource driven antimicrobials such as plant driven essential oils, organic acids, enzymes from animal sources and natural polymers have become increasingly attractive alternatives [7].

Essential oil is a hydrophobic liquid compartment obtained from various parts of plants such as flowers, seeds and stems [8]. Due to its aromatic characteristic, essential oil has long been used in the cosmetic and food industries as a flavoring agent. However, many studies have recognized other biological effects of essential oils such as antimicrobial, antidiabetic, and even anticancer activities [8]. Some essential oils possess antioxidant properties, which can have a positive effect on biological systems [9,10] as well as food production by preventing oxidation [11]. Among these biological functions, the antimicrobial properties of essential oils are popular and have thus become of interest as potential replacements for synthetic antimicrobials. Thyme, bay laurel, cinnamon, oregano, clove, and nutmeg exhibited antimicrobial activity against different Gram-negative and -positive bacteria—*Campylobacter jejuni*, *E. coli*, *S. typhimurium*, *Listeria monocytogenes* and *Staphylococcus aureus*—respectively [12,13]. Direct application of essential oils to food products such as beef also showed effective reduction of pathogens [14,15]. Essential oils have been shown to have advantages as natural antimicrobials. These oils’ various chemical compositions or single components at different concentrations have different inhibition mechanisms that can affect a variety of pathogens by changing membrane permeability, denaturing proteins and inhibiting enzymes [16]. Essential oils can also be used at low concentrations compared to synthetic antimicrobials and have shown effective antimicrobial activity against drug-resistant strains [16]. Moreover, essential oils did not affect existing beneficial intestinal bacteria such as *lactobacilli* and *bifidobacteria* [17]. These results suggest that essential oils would be potential candidates for development as natural antimicrobials, and the oils’ residues on foods could improve human health, leading to an alternative to the negative health impacts of synthetic preservatives [16].

White wormwood (*Artemisia herba-alba* Asso.) and rose-scented geranium (*Pelargonium capitatum* × *P. radens*), aromatic herbs have been used for medicinal purposes to treat stomach disorders, hypertension, inflammation and diabetes and have also been used in the cosmetic and food industries as flavoring agents [18,19,20]. Bay laurel (*Laurus nobilis* L.), an evergreen tree of the Luraceae family, is popular in the culinary and food industries as a spice and as a flavoring agent [21]. Although the antimicrobial and antioxidant activities of bay laurel essential oil have already been studied, its activities can vary depending on which extraction method is used as well as areas and conditions where it is grown [22,23]. The antimicrobial and antioxidant activities of white wormwood and rose-scented geranium essential oils remain to be explored.

The objective of the present study was to evaluate the antibacterial activities of commercial essential oils derived from white wormwood, rose-scented geranium and bay laurel against *S. typhimurium* and *E. coli* O157:H7, the most common pathogens found on fresh produce. To further explore whether these essential oils might also have antioxidant properties, their antioxidant activity was examined as well. Among these three essential oils, the one proven to be most effective in both prevention of *S. typhimurium* and *E. coli* O157:H7 and antioxidant activity was subjected to a sensory analysis in order to test for consumer acceptability of the oil’s usage on fresh produce.

## 2. Materials and Methods

### 2.1. Essential Oils

Essential oils derived from white wormwood (*Artemisia herba-alba* Asso.), rose-scented geranium (*Pelargonium capitatum* × *radens*), and leaves of bay laurel (*Laurus nobilis* L.) were purchased from New Directions Aromatics (Mississauga, Canada). The main components in each essential oil were provided from New Directions Aromatics (Mississauga, Canada) as follows: white wormwood essential oil consisted of 70% thujone; rose-scented geranium essential oil consisted of 16%–29% citronellol, 10%–25% geraniol, and 4%–8% linalool; bay laurel consisted of 30%–50% 1,8-cineol, 10%–20% linalool, 2.13% methyl eugenol and 0.01% eugenol. The oils were stored at 4 °C during the duration of the study.

### 2.2. Bacteria Strain and Enumeration

Freeze-dried *E. coli* O157:H7 (American Type Culture Collection; ATCC 700728) and *S. typhimurium* (ATCC 13311) were purchased from ATCC (Manassas, VA, USA). These isolates were transferred into a 5 mL brain heart infusion (BHI) broth, mixed and then incubated at 37 °C for 24 h. The bacterial suspension was then streaked on BHI agar plates. After incubation at 37 °C for 24 h, a single colony from each isolate was selected and inoculated into *Luria-Bertani* (LB) broth. The cells in the LB broth were incubated at 37 °C for 24 h.

### 2.3. Growth Over Time Assay

A growth over time assay was performed to test the antimicrobial activities of the essential oils [24]. In this study, 0%, 0.1%, 0.15%, 0.25%, 0.5% and 1% of essential oils (*v*/*v*) were prepared by dissolving essential oils into an LB broth mixed with 1% Tween-80. Bacteria of treated LB were individually inoculated to reach 2–3 log colony forming unit (CFU)/mL. The mixtures were then incubated at 37 ˚C for 8 h. Bacterial growth was monitored by measuring turbidity over time in 2, 4, 6 and 8 h intervals at 610 nm using a spectrophotometer (Genesys 10S UV-Vis, Thermo Scientific, Waltham, MA, USA). After 8 h of incubation, a 100 µL amount of individual bacterial suspension was serially diluted with peptone water. Appropriate dilutions were plated onto an LB agar plate and then incubated at 37 °C for 24 h to determine the final population.

### 2.4. Agar Diffusion Spot Assay

To determine bacterial susceptibility to essential oils, an agar diffusion spot assay was performed [25]. Fresh cultured bacteria were serially diluted up to four times by transferring 1 mL culture into 9 mL peptone water solution. The LB agar plates were inoculated with 0.5 mL of the fourth dilution of bacterial culture and then allowed to dry. Spots were formed by adding 20 µL of 0.1%, 0.2%, 0.3%, 0.4%, 0.5%, 0.6%, 0.7%, 0.8%, 1%, 3% and 8% of essential oils (*v*/*v*). Plates were incubated at 37 °C for 48 h. At 8 and 48 h incubation, bacteria growth was visually checked. The degree of bacterial inhibition was determined by no growth (−), some growth (−/+), or growth (+) in areas treated with essential oil. The minimum inhibition concentration (MIC) and minimum lethal concentration (MLC) were determined by the lowest concentration that completely inhibited bacterial growth after 8 and 48 h, respectively.

### 2.5. DPPH Free Radical Scavenging Activity

To examine the radical scavenging activity of the essential oils, 2,2-diphenyl-1-picrylhydrazyl (DPPH) stable free radical compound was used [26]. Briefly, 100 µL of 0.5%, 1%,10%, 20%, 30%, 50% and 100% of white wormwood and rose-scented geranium essential oils (*v*/*v*) or 0.0625%, 0.125%, 0.25%, 0.5%, 1%, 5% and 10% of bay laurel essential oil (*v*/*v*) were added to 900 µL of 70 µM DPPH. 100 µL of 10 mM butylated hydroxytoluene (BHT), a synthetic antioxidant, and methanol were used as a positive and negative control, respectively. After incubation at room temperature for 30 min, absorbance was measured at 517 nm using a spectrophotometer. The DPPH free radical scavenging activity was determined and expressed as % inhibition of essential oils in a DPPH free radical using the following formula:

(1) $$\%\;\text{scavenging activity}=\frac{\text{absorbance of an essential oil or a positive control}}{\text{absorbance of a negaive contraol}}\times 100.$$

In addition, EC_50_ was calculated to determine a concentration at which essential oils reduce the DPPH radical by 50% [27]. The experiment was conducted in quadruplicate.

### 2.6. Reducing Power Activity

The reducing powder of essential oils was determined by measuring a reduction of ferric to ferrous [28]. In addition, 100 µL of 10% or 100% essential oils (*v/v*) was mixed with 2.5 mL of 0.2 M phosphate buffer (pH 6.6) to which 2.5 mL of 1% (*v*/*v*) potassium ferric cyanide was then added. Mixtures were incubated for 20 min at 50 °C. At the end of the reaction, 2.5 mL of 10% (*v*/*v*) trichloroacetic acid was added and mixtures were centrifuged at 3000 rpm for 10 min. 2.5 mL of the upper layer solution was mixed with 2.5 mL of water and then 0.5 mL of 0.1% (*v*/*v*) ferric chloride was added. The absorbance was measured at 700 nm. All buffer and reaction mixtures were prepared with 9% tween-20 to completely dissolve essential oils. Ascorbic acid (5 mM) was used as a positive control. The experiment was performed in quadruplicate.

### 2.7. Total Antioxidant Activity

Total antioxidant activity was measured using the method described by Pan *et al*. [29]. A total of 100 µL of 0.0001% essential oils (*v*/*v*) dissolved in methanol were added to 1 mL of reaction solution (0.6 M sulphuric acid, 28 mM sodium phosphate and 4 mM ammonium molybdate), incubated in a water bath at 95 °C for 90 min and then cooled for 5 min. Absorbance was measured at 695 nm, and 5 mM ascorbic acid and methanol were used as a positive and a negative control, respectively. All tests were performed in quadruplicate.

### 2.8. Sensory Analysis

A sensory analysis was performed on fresh produce (tomatoes, cantaloupe and spinach) that were hand-sprayed with 0.4% (*v*/*v*) rose-scented geranium essential oil [30]. Fresh produce as a control was sprayed with tap water. Each sample was allocated a three-digit random number to avoid bias by the panelists. The panel consisted of 14 assessors (six female and eight male) with previous experience in sensory evaluation. To determine the acceptability of the fresh produce, a 9-point hedonic scale was used where 9 = like extremely, 8 = like very much, 7 = like moderately, 6 = like slightly, 5 = neither like nor dislike, 4 = dislike slightly, 3 = dislike moderately, 2 = dislike very much, and 1 = dislike extremely was used. All panelists were asked to evaluate for color, browning, vegetable aroma, off-odor, texture and overall acceptability.

### 2.9. Statistical Analysis

Data were expressed as means ± standard deviation (SD). One-way ANOVA was used for statistical analysis with a Tukey’s test to determine difference among comparisons in *R* Statistical software (version 2.15.2). Difference was considered significant at *p* < 0.05.

## 3. Results and Discussion

### 3.1. Effect of Essential Oils on the Growth of S. typhimurium and E. coli O157:H7

**Figure 1:**
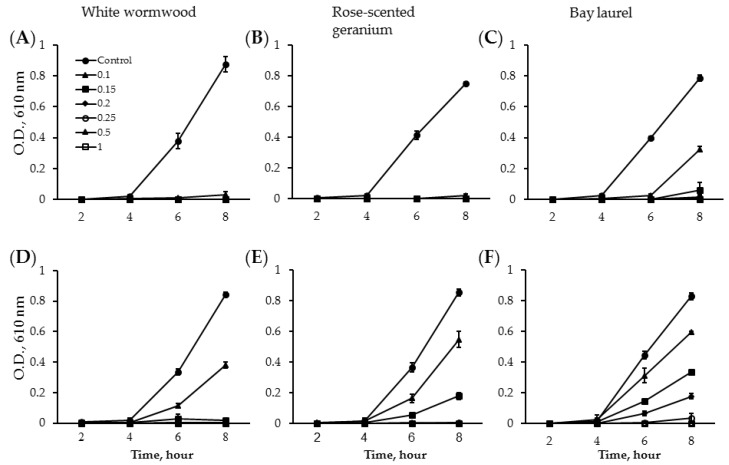
Inhibitory effect of white wormwood, rose-scented geranium and bay laurel essential oils on growth of *S. typhimurium* and *E. coli* O157:H7. (**A**–**C**) represents the growth of *S. typhimurium* and (**D**–**F**) represent the growth of *E. coli* O157:H7 in the presence of different concentrations of white wormwood (**A** and **D**), rose-scented geranium (**B** and **E**) and bay laurel (**C** and **F**) essential oils in LB broth during 8 h incubation (37 °C) by measuring O.D. (610 nm). The experiment was performed in triplicate. The data was presented as mean ± SD.

To examine the effects of white wormwood, rose-scented geranium and bay laurel essential oils on the growth of *S*. *typhimurium* and *E. coli* O157:H7, bacterial growth was monitored by measuring turbidity as optical density (O.D.) in 2 h intervals during 8 h of incubation with the essential oils (Figure 1). *S*. *typhimurium* and *E. coli* O157:H7 showed detectable populations after 4 h incubation, and then their growth sharply increased over the next 4 h (Figure 1). At the end of 8 h of incubation, O.D. from both bacterial cultures exhibited 0.75–0.88. The addition of 0.1% white wormwood and rose-scented geranium essential oils resulted in a 97% reduction in the O.D. of *S*. *typhimurium*, whereas 0.15% white wormwood and 0.2% rose-scented geranium essential oils were required to produce a similar reduction in *E. coli* O157:H7 (Figure 1A,B,D,E). Among the three essential oils, bay laurel essential oil exhibited the lowest bacterial inhibition (Figure 1C,F). Upon completion of the growth over time assay, cells were subjected to a survival count (Table 1). In the control, *S*. *typhimurium* and *E. coli* O157:H7 were 8.89–9.68 log CFU/mL. With the addition of 0.1% white wormwood and rose-scented geranium essential oils, the growth of *S*. *typhimurium* was reduced to 2.87 ± 0.43 and 1.72 ± 0.22 log CFU/mL as indicated by a 70% and 81% inhibition, respectively, whereas 0.15% bay laurel essential oils blocked 64% of bacterial growth (Table 1). The *S*. *typhimurium* population was reduced to below detectable levels by concentrations of more than 0.1% white wormwood, rose-scented geranium and 0.2% bay laurel essential oil (Table 1). White wormwood essential oil at 0.2% showed a 66% reduction in *E. coli* O157:H7, indicated by 3.04 ± 0.14 log CFU/mL (Table 1). The bacterial population was reduced to below detectable levels at concentrations higher than 0.2%. Rose-scented geranium exhibited a similar inhibition at slightly higher concentrations compared to white wormwood essential oil, whereas 0.25% bay laurel essential oil showed a 54% reduction in *E. coli* O157:H7, supported by 4.17 ± 0.31 log CFU/mL. White wormwood at 0.25% and bay laurel at 0.5% reduced the *E. coli* O157:H7 population to below detectable levels.

**Figure 2:**
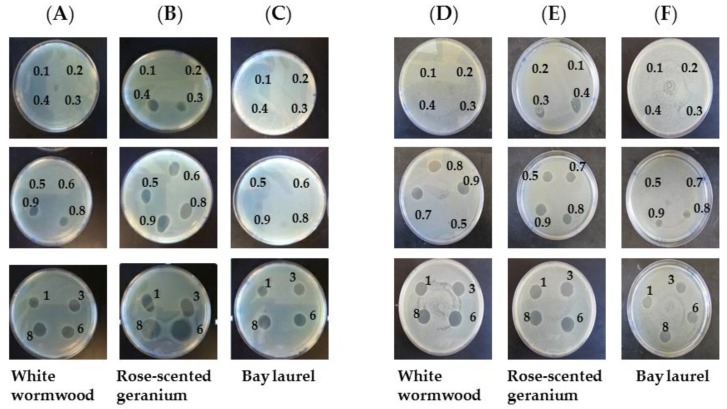
A representative image obtained by results of agar diffusion spot assay. (**A**–**C**) *S*. *typhimurium* and (**D**–**F**) *E. coli* O157:H7 were streaked in each plate and then different concentrations of white wormwood (**A** and **D**), rose-scented geranium (**B** and **E**) and bay laurel (**C** and **F**) essential oils were spot in the plate. After 48 h incubation, a zone of inhibition was observed.

To determine the lowest concentration that completely inhibited *S. typhimurium* and *E. coli* O157:H7 growth, essential oils were directly applied to agar plates inoculated with *S. typhimurium* and *E. coli* O157:H7. After 8 h of incubation, some colonies of *S. typhimurium* and *E. coli* O157:H7 were observed on a spotted area with 0.8%, 0.3% and 1% and 0.4%, 0.3% and 0.6% of white wormwood, rose-scented geranium and bay laurel essential oils, respectively (Table 2 and Figure 2). In contrast, 0.9%, 0.4% and 3% and 0.5%, 0.4% and 0.8% of white wormwood, rose-scented geranium and bay laurel essential oils completely inhibited visual growth of *S. typhimurium* and *E. coli* O157:H7, indicated as MIC in which no colonies were detected on the spotted area. The concentration at which bacteria grew after 48 h incubation was determined as MIC (Table 2 and Figure 2). *S. typhimurium* grew back in areas treated with less than 1% white wormwood after 48 h. This indicated that the MLC of its essential oil was determined to be 3%. Rose-scented geranium and bay laurel essential oils showed the same concentrations for MLC as their MIC against *S. typhimurium*. After 48 h, *E. coli* O157:H7 grew back at the MIC of three essential oils as well. No colony was observed in areas treated with 0.6% and 0.5% white wormwood and rose geranium, respectively, as indicated by MLC. However, *E. coli* O157:H7 grew in areas treated with bay laurel essential oil up to 0.9%. This indicated that the MLC of bay laurel was 1% with no colony in the area.

All of the essential oils used in this study showed significant antibacterial activity, with the variation depending on both types and concentrations of essential oil. Although white wormwood essential oil inhibited the growth of *E. coli* O157:H7 at a slightly lower concentration than rose-scented geranium essential oil, white wormwood and rose-scented geranium essential oil exhibited similar growth inhibition against *S. typhimurium* in the growth over time assay. All essential oils that were tested with this method exhibited a greater sensitivity to *S. typhimurium* than to *E. coli* O157:H7. In contrast, white wormwood and bay laurel essential oils showed less sensitivity to *S. typhimurium* than *E. coli* O157:H7 in the agar diffusion spot method. Compared to white wormwood and bay laurel essential oils, rose-scented geranium showed the highest antimicrobial activity among the three essential oils, confirmed by MIC and MLC against *S. typhimurium* and *E. coli* O157:H7 at 0.4% and 0.4% and 0.4% and 0.5%, respectively. It is notable that the similar MIC and MLC of rose-scented geranium indicated the effectiveness of rose-scented geranium essential oil at inhibiting both common pathogens at the same concentration levels. Boukhatem *et al*. [31] reported that rose-scented geranium essential oil had antimicrobial activity against *E. coli* but no inhibition effect against *S. typhimurium* when tested using a disk diffusion method. In a study by Sbayou *et al*. [32], MIC and MLC of white wormwood essential oil against *E. coli* O157:H7 was approximately 0.125%, which was lower than our current data. Although bay laurel essential oil showed the lowest antimicrobial activities among three essential oils in the present study, other studies showed higher values for MIC and MLC of bay laurel essential oil against the same pathogens [31,33]. This difference may be due to several factors such as variation in the percentage of main components, different extraction methods, or different methods for evaluating the antibacterial activities.

### 3.2. Antioxidant Effect of Essential Oils

**Figure 3:**
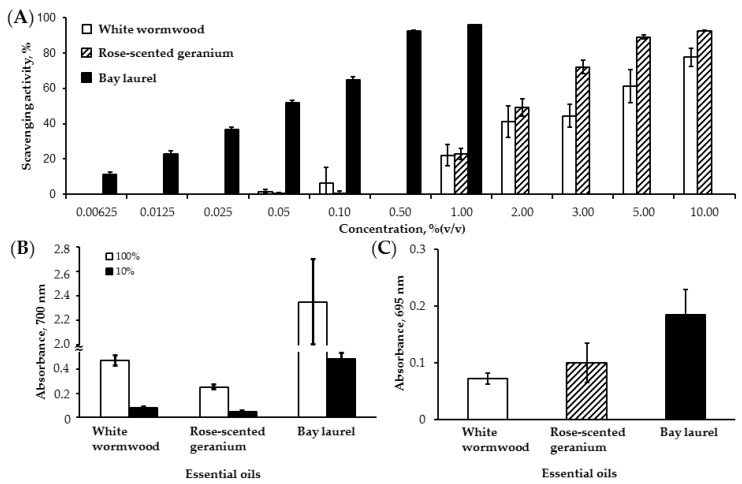
Antioxidant activity of white wormwood, rose-scented geranium and bay laurel essential oils. (**A**) DPPH radical scavenging activity; (**B**) reducing power activity; (**C**) total antioxidant activity. Each experiment was performed in quadruplicate. The data was presented as mean ± SD.

Next, antioxidant effects of essential oils from white wormwood, rose-scented geranium and bay laurel were examined. Figure 3A shows the radical scavenging activities of the essential oils using DPPH, a stable free radical. Although white wormwood and rose-scented geranium essential oils exhibited similar radical scavenging activity, rose-scented geranium showed higher activity than that of white wormwood at concentrations higher than 3% (Figure 3A). Because of the strong scavenging activity of bay laurel essential oil, measurement was performed at lower concentrations. Compared to the other oils, 1% bay laurel essential oil showed a 96% DPPH radical scavenging activity. This was confirmed by EC_50_ values, 2.33 ± 0.47, 1.85 ± 0.20, and 0.04 ± 0.005 of white wormwood, rose-scented geranium and bay laurel essential oils, respectively. The electron donating capacity of essential oils was accessed using a reducing power assay (Figure 3B). Bay laurel essential oil exhibited a higher reducing power than white wormwood and rose geranium. Consistent with this, bay laurel essential oil also exhibited a higher total antioxidant activity than the others. Rose-scented geranium essential oil showed a higher total antioxidant activity than white wormwood, but this difference was not statistically significant.

Essential oils have shown antioxidant properties that improve health as well as food quality [34]. Consistent with our present data, bay laurel essential oil showed strong radical scavenging activity, electron donating capacity and total antioxidant activity [35,36,37,38]. Bay laurel essential oil used in a present study contained 1.8-cineole, linalool, methyl eugenol and eugenol. The primary components of morocco bay laurel essential oil that were extracted using a stream distillation method were 1,8-cineole, 2-carene, trans-ocimene and sabinene [37]. In the study of Saab *et al*. [39], 1.8-cineole, 1-p-menthen-8-ethyl acetate, linalool and sabinene were indicated as main components of essential oil obtained from laurel leaves. The strong antioxidant property of bay laurel essential oil may be due to the presence of its primary components 1,8-cineole and linalool. However, the individual components did not show strong antioxidant activity [35]. This would suggest that the strong activity of bay laurel essential oil may be due to the presence of other minor components, or the synergistic effects between the major and minor components or phenolic compounds that are possibly extracted at the same time during the essential oils extraction process. Essential oil obtained from white wormwood growing in Tunisia using a hypodistillation method showed antioxidant activity [40]. One of five chemical classes identified in the essential oil was monoterpenes, which includes thujone (approximately 10%) [40]. White wormwood essential oil that was tested in a present study includes 70% thujone. Guerrini *et al.* [41] reported that rose-scented geranium essential oil had lower antioxidant activity than thyme essential oil. This showed similar main chemical composition, but higher concentrations of citronellol (32.71%) and geraniol (19.58%) compared to the essential oil used in a present study [41]. However, methanol extract obtained from white wormwood exhibited stronger scavenging and reducing power activities due to phenolic compositions [42]. There can be variations in the chemical composition of essential oils due to differences in geographic origin of the plant and temperature and the length of growing seasons. These variations might influence the antioxidant and antimicrobial activities of the essential oils.

### 3.3. Sensory Evaluation

Based on the antimicrobial and antioxidant effects of essential oils in Figure 1, Figure 2 and Figure 3 and Table 1, rose-scented geranium exhibited the highest antimicrobial and antioxidant effects and was thus selected for sensory evaluation on fruits and vegetables. Table 3 shows the results of the sensory evaluation of whole tomatoes, cantaloupe and spinach sprayed with and without rose-scented geranium essential oils. Statistical analysis indicated that there was no significant difference (*p* > 0.05) between the essential oil-treated and water-sprayed groups with regard to aroma, color, texture, browning, off-odor and overall acceptability. This result would suggest that rose-scented geranium (0.4%, *v*/*v*) could be an attractive antimicrobial to prevent contamination from both *S. typhimurium* and *E. coli* O157:H7 on fresh produce. In addition, the antioxidant properties of rose-scented geranium residue could provide a positive health effect.

## 4. Conclusions

Rose-scented geranium essential oil exhibited the highest antimicrobial activity against both *S. typhimurium* and *E. coli* O157:H7 at equal concentrations. The antioxidant properties of this essential oil could also provide a health benefit to consumers in contrast to the negative health issues associated with the consumption of synthetic antimicrobials. Thus, rose-scented geranium essential oil could be a promising candidate for development as an alternative natural antimicrobial to prevent contamination by *S. typhimurium* and *E. coli* O157:H7 in food products. The results of this study demonstrated the antimicrobial activity of three essential oils against foodborne pathogens grown in laboratory media. However, further study is warranted in order to explore the effectiveness of these essential oils alone or in combination with various types of physical treatments on foodborne pathogens, in order to compare the effectiveness of these oils in inhibiting foodborne pathogens.

## Figures and Tables

**Table 1:** Final population of *S. typhimurium* and *E. coli* O157:H7 in LB broth in the presence of essential oils at different concentrations (%, *v*/*v*) after 8 h of incubations (37 °C).

	*S. typhimurium*			*E. coli* O157:H7		
Concentrations (%, *v*/*v*)	White wormwood	Rose-scented geranium	Bay laurel	White wormwood	Rose-scented geranium	Bay laurel
Control	9.68^Aa^ ± 0.14	9.15^Ab^ ± 0.12	9.34^Ac^ ± 0.18	8.89^Aa^ ± 0.28	9.08^Aa^ ± 0.27	8.99^Aa^ ± 0.20
0.1	2.87^Ba^ ± 0.43	1.72^Ba^ ± 0.22	7.01^Bb^ ± 0.42	7.02^Ba^ ± 0.23	7.81^Bab^ ± 0.42	8.06^Bb^ ± 0.28
0.15	BDL	BDL	3.38^C^ ± 0.33	3.04^Ca^ ± 0.14	5.05^Cb^ ± 0.30	6.30^Cc^ ± 0.21
0.2	BDL	BDL	BDL	BDL	3.38^Da^ ± 0.25	5.17^Db^ ± 0.19
0.25	BDL	BDL	BDL	BDL	BDL	4.17^E^ ± 0.31
0.5	BDL	BDL	BDL	BDL	BDL	BDL
1	BDL	BDL	BDL	BDL	BDL	BDL

Averages with different upper case letters (A, B, C, D, and E) in the same column are significantly different (*p* < 0.05); Averages with different lower case letters (a, b, c and d) in the same row and for each strain are significantly different (*p* < 0.05); BDL: Below detectable level.

**Table 2:** Inhibitory effect of essential oils against *S. typhimurium* and *E. coli* O157:H7 after 8 and 48 h incubation on LB agar plate.

Hour	Concentration (%, *v*/*v*)	0.1	0.2	0.3	0.4	0.5	0.6	0.8	0.9	1	3	6	8
*S. typhimurium*													
8	White wormwood	+	+	+	+	+	+	+/−	−	−	−	−	−
	Rose-scented geranium	+	+	+/−	−	−	−	−	−	−	−	−	−
	Bay laurel	+	+	+	+	+	+	+	+/−	+/−	−	−	−
48	White wormwood	+	+	+	+	+	+	+/−	+/−	+/−	−	−	−
	Rose-scented geranium	+	+	+/−	−	−	−	−	−	−	−	−	−
	Bay laurel	+	+	+	+	+	+	+	+	+/−	−	−	−
*E. coli* O157:H7													
8	White wormwood	+	+	+	+/−	−	−	−	−	−	−	−	−
	Rose-scented geranium	+	+	+/−	−	−	−	−	−	−	−	−	−
	Bay laurel	+	+	+	+	+	+/−	−	−	−	−	−	−
48	White wormwood	+	+	+	+	+/−	−	−	−	−	−	−	−
	Rose−scented geranium	+	+	+/−	+/−	−	−	−	−	−	−	−	−
	Bay laurel	+	+	+	+	+	+	+/−	+/−	−	−	−	−

(−) no growth; (±) some growth; (+) growth.

**Table 3:** Effect of rose-scented geranium essential oil on the sensory characteristics of fresh produce.

**Whole Tomatoes**						
	Aroma	Off-odor	Color	Browning	Texture	Overall acceptability
Control	7.14 ± 2.46^a^	5.18 ± 1.9^a^	7.14 ± 1.15^a^	5.36 ± 0.95^a^	6.79 ± 1.68^a^	6.68 ± 2.23^a^
RG*	5.75 ± 2.23^a^	4.82 ± 1.76^a^	6.79 ± 1.40^a^	5.14 ± 1.04^a^	6.57 ± 1.54^a^	5.68 ± 2.08^a^
*t*-value	1.57	0.51	0.74	0.57	0.35	1.23
*f*-value	0.128	0.615	0.467	0.575	0.728	0.231
**Cantaloupes**						
	Aroma	Off-odor	Color	Browning	Texture	Overall acceptability
Control	7.29 ± 1.03^a^	5.96 ± 1.29^a^	6.39 ± 1.86^a^	5.75 ± 1.64^a^	6.07 ± 1.96^a^	6.50 ± 1.73^a^
RG*	6.32 ± 1.79^a^	5.50 ± 1.77^a^	6.79 ± 1.63^a^	5.68 ± 1.19^a^	6.61 ± 1.59^a^	6.21 ± 1.67^a^
*t*-value	1.74	0.79	0.59	0.13	0.79	0.44
*f*-value	0.093	0.434	0.55	0.896	0.435	0.661
**Spinach Leaves**										
	Aroma	Off-odor	Color	Browning	Texture	Overall acceptability
Control	6.43 ± 2.04^a^	5.86 ± 1.54^a^	7.46 ± 1.15^a^	5.36 ± 1.79^a^	6.86 ± 1.59^a^	7.36 ± 1.17^a^
RG*	6.04 ± 1.93^a^	5.43 ± 1.05 ^a^	7.54 ± 1.28^a^	5.29 ± 1.61^a^	6.93 ± 1.20^a^	6.86 ± 1.63^a^
*t*-value	0.52	0.86	0.16	0.11	0.13	0.93
*f*-value	0.605	0.397	0.878	0.913	0.896	0.360

Mean values sharing same letters are non-significant to each other; ^a^ a sample with score less than 5 in any of the sensory attributes was considered unacceptable; * RG is rose-scented geranium essential oil.

## References

[1] Scallan E., Hoekstra R.M., Angulo F.J., Tauxe R.V., Widdowson M.A., Roy S.L., Jones J.L., Griffin P.M. (2011). Foodborne illness acquired in the united states-major pathogens. Emerg. Infect. Dis..

[2] Rahal E.A., Kazzi N., Nassar F.J., Matar G.M. (2012). *Escherichia coli* O157:H7—Clinical aspects and novel treatment approaches. Front. Cell Infect Microbiol..

[3] Golberg D., Kroupitski Y., Belausov E., Pinto R., Sela S. (2011). *Salmonella typhimurium* internalization is variable in leafy vegetables and fresh herbs. Int. J. Food Microbiol..

[4] Anand S.P., Sati N. (2013). Artificial preservatives and their harmful effects: Looking toward nature for safer alternatives. Int. J. Pharm. Sci. Res..

[5] Rafii F., Sutherland J.B., Cerniglia C.E. (2008). Effects of treatment with antimicrobial agents on the human colonic microflora. Ther. Clin. Risk Manag..

[6] Lai E.P., Iqbal Z., Avis T.J. (2016). Combating antimicrobial resistance in foodborne microorganisms. J. Food Prot..

[7] Lucera A., Costa C., Conte A., Del Nobile M.A. (2012). Food applications of natural antimicrobial compounds. Front. Microbiol..

[8] Bakkali F., Averbeck S., Averbeck D., Idaomar M. (2008). Biological effects of essential oils—A review. Food Chem. Toxicol..

[9] Loizzo M.R., Menichini F., Tundis R., Bonesi M., Conforti F., Nadjafi F., Statti G.A., Frega N.G., Menichini F. (2009). *In vitro* biological activity of salvia leriifolia benth essential oil relevant to the treatment of alzheimer’s disease. J. Oleo. Sci..

[10] Loizzo M.R., Ben Jemia M., Senatore F., Bruno M., Menichini F., Tundis R. (2013). Chemistry and functional properties in prevention of neurodegenerative disorders of five cistus species essential oils. Food Chem. Toxicol..

[11] Tongnuanchan P., Benjakul S. (2014). Essential oils: Extraction, bioactivities, and their uses for food preservation. J. Food Sci..

[12] Dorman H.J., Deans S.G. (2000). Antimicrobial agents from plants: Antibacterial activity of plant volatile oils. J. Appl. Microbiol..

[13] Smith-Palmer A., Stewart J., Fyfe L. (1998). Antimicrobial properties of plant essential oils and essences against five important food-borne pathogens. Lett. Appl. Microbiol..

[14] Cutter C.N. (2000). Antimicrobial effect of herb extracts against escherichia coli o157: H7, listeria monocytogenes, and salmonella typhimurium associated with beef. J. Food Prot..

[15] Kotzekidou P., Giannakidis P., Boulamatsis A. (2008). Antimicrobial activity of some plant extracts and essential oils against foodborne pathogens in vitro and on the fate of inoculated pathogens in chocolate. LWT-Food Sci. Technol..

[16] Nazzaro F., Fratianni F., De Martino L., Coppola R., Vincenzo D.F. (2013). Effect of essential oils on pathogenic bacteria. Pharmaceuticals (Basel).

[17] Si W., Gong J., Tsao R., Zhou T., Yu H., Poppe C., Johnson R., Du Z. (2006). Antimicrobial activity of essential oils and structurally related synthetic food additives towards selected pathogenic and beneficial gut bacteria. J. Appl. Microbiol..

[18] Moufid A., Eddouks M. (2012). Artemisia herba alba: A popular plant with potential medicinal properties. Pak. J. Biol. Sci..

[19] Boukhatem M.N., Kameli A., Ferhat M.A., Saidi F., Mekarnia M. (2013). Rose geranium essential oil as a source of new and safe anti-inflammatory drugs. Libyan. J. Med..

[20] Setzer W.N. (2009). Essential oils and anxiolytic aromatherapy. Nat. Prod. Commun..

[21] Fang F., Sang S., Chen K.Y., Gosslau A., Ho C., Rosen R.T. (2005). Isolation and identification of cytotoxic compounds from bay leaf (*Laurus nobilis*). Food Chem..

[22] Marzouki H., Khaldi A., Marongiu B., Piras A., Harzallah-Skhiri F. (2011). Chemical polymorphism of essential oils from populations of laurus nobilis grown on tunisia, algeria and france. Nat. Prod. Commun..

[23] Dias M.I., Barros L., Dueñas M., Alves R.C., Oliveira M.B., Santos-Buelga C., Ferreira I.C. (2014). Nutritional and antioxidant contributions of *Laurus nobilis* L. Leaves: Would be more suitable a wild or a cultivated sample?. Food Chem..

[24] Hayek S.A., Ibrahim S.A. (2012). Antimicrobial activity of xoconostle pears (*Opuntia matudae*) against escherichia coli o157:H7 in laboratory medium. Int. J. Microbiol..

[25] Awaisheh S.S., Al-Nabulsi A.A., Osaili T.M., Ibrahim S., Holley R. (2013). Inhibition of cronobacter sakazakii by heat labile bacteriocins produced by probiotic lab isolated from healthy infants. J. Food. Sci..

[26] Yu L., Haley S., Perret J., Harris M., Wilson J., Qian M. (2002). Free radical scavenging properties of wheat extracts. J. Agric. Food Chem..

[27] Alexander B., Browse D.J., Reading S.J., Benjamin I.S. (1999). A simple and accurate mathematical method for calculation of the ec50. J. Pharmacol. Toxicol. Methods.

[28] Pan Y., He C., Wang H., Ji X., Wang K., Liu P. (2010). Antioxidant activity of microwave-assisted extract of buddleia officinalis and its major active component. Food Chem..

[29] Pan Y., Wang K., Huang S., Wang H., Mu X., He C., Ji X., Zhang J., Huang F. (2008). Antioxidant activity of microwave-assisted extract of longan (*Dimocarpus longan* Lour.) peel. Food Chem..

[30] Plemmons L.E., Resurreccion A.V.A. (1998). A warm-up sample improves reliability of responses in descriptive analysis. J. Sens. Stud..

[31] Boukhatem M.N., Kameli A., Saidi F. (2013). Essential oil of algerian rose-scented geranium (*Pelargonium graveolens*): Chemical composition and antimicrobial activity against food spoilage pathogens. Food Control.

[32] Sbayou H., Ababou B., Boukachabine K., Manresa A., Zerouali K., Amghar S. (2014). Chemical composition and antibacterial activity of artemisia herba-alba and mentha pulegium essential oils. J. Life Sci..

[33] Da Silveira S.M., Cunha A., Scheuermann G.N., Secchi F.L., Vieira C.R.W. (2012). Chemical composition and antimicrobial activity of essential oils from selected herbs cultivated in the south of brazil against food spoilage and foodborne pathogens. Cienc. Rural.

[34] Hamidpour M., Hamidpour R., Hamidpour S., Shahlari M. (2014). Chemistry, pharmacology, and medicinal property of sage (salvia) to prevent and cure illnesses such as obesity, diabetes, depression, dementia, lupus, autism, heart disease, and cancer. J. Tradit. Complement. Med..

[35] Lee S., Umano K., Shibamoto T., Lee K. (2005). Identification of volatile components in basil (*Ocimum basilicum* L.) and thyme leaves (*Thymus vulgaris* L.) and their antioxidant properties. Food Chem..

[36] Politeo O., Jukić M., Miloš M. (2007). Chemical composition and antioxidant activity of free volatile aglycones from laurel (*Laurus nobilis* L.) compared to its essential oil. Croat. Chem. Acta..

[37] Cherrat L., Espina L., Bakkali M., García-Gonzalo D., Pagán R., Laglaoui A. (2014). Chemical composition and antioxidant properties of *Laurus nobilis* L. And *Myrtus communis* L. Essential oils from morocco and evaluation of their antimicrobial activity acting alone or in combined processes for food preservation. J. Sci. Food Agric..

[38] Ekren S., Yerlikaya O., Tokul H.E., Akpınar A., Accedil M. (2013). Chemical composition, antimicrobial activity and antioxidant capacity of some medicinal and aromatic plant extracts. Afr. J. Microbiol. Res..

[39] Saab A.M., Tundis R., Loizzo M.R., Lampronti I., Borgatti M., Gambari R., Menichini F., Esseily F., Menichini F. (2012). Antioxidant and antiproliferative activity of *Laurus nobilis* L. (lauraceae) leaves and seeds essential oils against k562 human chronic myelogenous leukaemia cells. Nat. Prod. Res..

[40] Zouari S., Zouari N., Fakhfakh N., Bougatef A., Ayadi M.A., Neffati M. (2010). Chemical composition and biological activities of a new essential oil chemotype of tunisian *Artemisia herba alba* asso. J. Med. Plants Res..

[41] Guerrini A., Muzzoli M., Romagnolid C., Antognoni F. (2011). Chemical characterization (GC/MS and NMR fingerprinting) and bioactivities of south-african *Pelargonium capitarum* (L.) L’HER. Chem. Biodiv..

[42] Khlifi D., Sghaier R.M., Amouri S., Laouini D., Hamdi M., Bouajila J. (2013). Composition and anti-oxidant, anti-cancer and anti-inflammatory activities of artemisia herba-alba, *Ruta chalpensis* L. And *Peganum harmala* L.. Food Chem. Toxicol..

